# Supplementary material for: The Se–S Bond Formation in the Covalent Inhibition Mechanism of SARS-CoV-2 Main Protease by Ebselen-like Inhibitors: A Computational Study
Source: Int J Mol Sci. 2021 Sep 10;22(18):9792. doi: 10.3390/ijms22189792 (PMC8467846; doi:10.3390/ijms22189792)
Supplement: Supplementary file 1 [file ijms-22-09792-s001.zip › ijms-1359625-supplementary.pdf]

Supplementary Materials

# The Se-S bond formation in the covalent inhibition mechanism of SARS-CoV-2 main protease by ebselen-like inhibitors. A computational study

**Angela Parise[1,2], Isabella Romeo[1], Nino Russo[1] and Tiziana Marino [1*]**

[1] Dipartimento di Chimica e Tecnologie Chimiche, Università della Calabria, Via Pietro Bucci, 87036 Arcavacata di Rende, CS, Italy
[2] Université Paris-Saclay, CNRS, Institut de Chimie Physique UMR8000, Orsay, France
* Correspondence: tiziana.marino65@unical.it

**Table of Contents**

Molecular Dynamics procedure

Molecular Dynamics analysis

**Scheme S1.** Proposed mechanism for the covalent inhibition of $M^{pro}$

**Figure S1.** Superposition of crystallographic structures

**Figure S2.** MD most populated structures

**Figure S3.** RMSD analysis

**Figure S4.** RMSF analysis

**Figure S5.** Pocket volume cavity

**Figure S6.** SASA

**Figure S7.** Catalytic dyad distance

**Figure S8.** Salt bridge

**Figure S9.** RDF water

**Figure S10.** Docking protocol setting

**Figure S11.** Best docked poses

**Figure S12.** Specific distances $M^{pro}$ –EBS

**Figure S13.** Specific distances $M^{pro}$ –EBS-OH

**Figure S14.** Optimized structures of HIE stationary points

**Figure S15.** Free energy profiles of $M^{pro}$ HIE

**Table S1.** pKa values

**Table S2.** Donor-Acceptor H-bond $M^{pro}$ HID

**Table S3.** Donor-Acceptor H-bond $M^{pro}$ HIE

**Table S4.** RMSD-based clustering

**Table S5.** Docking scores

**Table S6.** NBO charges of EBS and EBS-OH

**Table S7.** NBO charges by atoms ($M^{pro}$ HID – EBS)

**Table S8.** NBO charges by atoms ($M^{pro}$ HIE – EBS)

**Table S9.** NBO charges by atoms ($M^{pro}$ HID – EBS-OH)

**Table S10.** Energy values corrected including free energy (kcal/mol) in gas phase for the three used basis sets for the inhibition phase

**References**

**Molecular Dynamics procedure**

The crystal structure of main protease bound to potent broad-spectrum non-covalent inhibitor X77 with PDB code 6W63[1] were used as starting point. The structure is characterized by one chain of 306 amino acids. The apo-enzyme was obtained by removing the inhibitor from the crystallographic structure. The structure thus obtained was superimposed on other crystallographic structures (PDB codes: 6LU7[2] and 6Y2F[3]) with an RMSD value lower than 1 Å. (Figure S1).

The absent hydrogen atoms were added using H++ server[4] to calculate the protonation states of titratable residues at pH 7.4 (Table S1). The protonation states of the catalytic residue neutral His41, with the hydrogen on the side chain Nε (HIE) and Nδ (HID), have been considered. 300 ns MDs were performed for apo-$M^{pro}$, in the two protonation states (HIE and HID).

Each system has been solvated in an orthorhombic box with a buffer of 10 Å, using TIP3P water model. $Na^+$ ions have been added to ensure zero total charge. The solvated structure was minimized in stages using AMBER 16.[5] In particular, water molecules and counterions were minimized over 2500 steps of steepest descent and 2500 steps of conjugate gradient minimization with a 50 kcal $mol^{-1}$ $Å^{-2}$ constraint on the solute. Subsequentially, hydrogen atoms were minimized over 5000 steps of steepest descent and 5000 steps of conjugate gradient with a 50 kcal $mol^{-1}$ $Å^{-2}$ constraint on all heavy atoms. Next, 5000 steps of steepest descent and 5000 steps of conjugate gradient minimization was performed, with 50 kcal $mol^{-1}$ $Å^{-2}$ constraint on protein backbone. Finally, the whole system has been released with 3000 and 6000 steps of unconstrained steepest descent and conjugate gradient respectively. Each stage of minimization was run until the root-mean-square of the Cartesian gradient was low than $10^{-4}$ kcal $mol^{-1}$ $Å^{-1}$. Then a progressive heating phase was carried out from 0 to 310 K for 0.5 ns using the Langevin thermostat ( g = 2.0 ) in NVT ensemble. The production phase consist of 300 ns of MDs using FF14SB force field[6] under the following conditions: integration step of 2 fs coupling SHAKE algorithm; NPT ensemble at 1 bar pressure using the Berendsen barostat[7] with a time constant τp = 2.0 ps. The Particle mesh Ewald summation method[8] has been employed for the electrostatic potential long-range interactions with a 12 Å cut-off distance.

In order to select different representative conformations of the system, root-mean square deviation (RMSD) based clustering of the whole trajectory has been performed using the an agglomerative bottom-up approach (hieragglo algorithm) available in Amber16 tools. After removing overall rotations and translations by RMS-fitting the C$\alpha$ atoms' positions of the trajectory, the average linkage clustering algorithm has been applied, identifying 10 representative conformations of the protein (Figure S2 and Table S2).

Afterwards, the representative structures with the highest percentage of population (Table S2) were considered for the further molecular docking studies, performed by using AutoDock (version 4.2).[9]

The ligands (EBS and EBS-OH) and the SARS-CoV-2 M$^{pro}$ models, apo-HID and apo-HIE, were processed using the AutoDock Tools [9] to obtain the PDBQT (Protein Data Bank, Partial Charge (Q), & Atom Type (T)) coordinate files containing the information needed by AutoGrid and AutoDock, namely polar hydrogen atoms, partial charges, correct atom types, and information on the articulation of flexible molecules. In order to verify if the adopted docking procedure is adequate, we have performed the docking of X77 inhibitor present in the used crystallographic structure. The results, reported in Figure S10 (as docking protocol setting) return a good superposition with a RMSD of 1.91 Å. In particular, Gasteiger-Marsili charges[10] were loaded in ADT (Auto Dock Tools). The center of the grid box locates on the sulfur atom of Cys145, and a dimension box of 70x70x70 Å has been chosen to abundant cover the active site. For ligand conformational searching the Lamarckian genetic algorithm (LGA)[11] has been used. The docking conditions were as follows: 10 independent runs, population size of 150, random starting position and conformation, local search rate of 0.6 and 2500000 energy evaluations. Final docked poses have been clustered using RMSD tolerance of 0.5 Å (Table S5).

The best docking pose was chosen according to the most negative docking score (Figure S11).

In order to obtain ebselen-like compound (EBS and EBS-OH) parameters gas phase geometry optimization has been carried out using B3LYP/6-31G*. Atomic charges are derived by fitting the electrostatic potential according to the Merz−Singh−Kollman scheme,[12] using the RESP fitting procedure. Antechamber and parmchk modules of Amber16 has been used for generating preparatory files to perform MM relaxation of the complexes HID-EBS, HIE-EBS and HID-EBS-OH.

**Molecular Dynamics analysis**

Root-mean-square deviation (RMSD) of the protein M$^{pro}$ HID and M$^{pro}$ HIE backbone, RMSD of the active site, root mean square fluctuation (RMSF), solvent accessible surface area (SASA), radial distribution function (RDF), hydrogen (H)-bond analysis and RMSD-based clustering MD simulation were performed using cpptraj module of AmberTools 16.[5] Electrostatic calculations were performed with the Adaptive Poisson-Boltzmann Solver (APBS).[13] Volume cavity of the binding pocket was performed with CAVER 2.0 Analyst[14] , a very rough estimation of the volume. The volume of the pocket ,in cubic Å, is computed by random sampling the bounding box of filling balls with a radius of 5 Å.

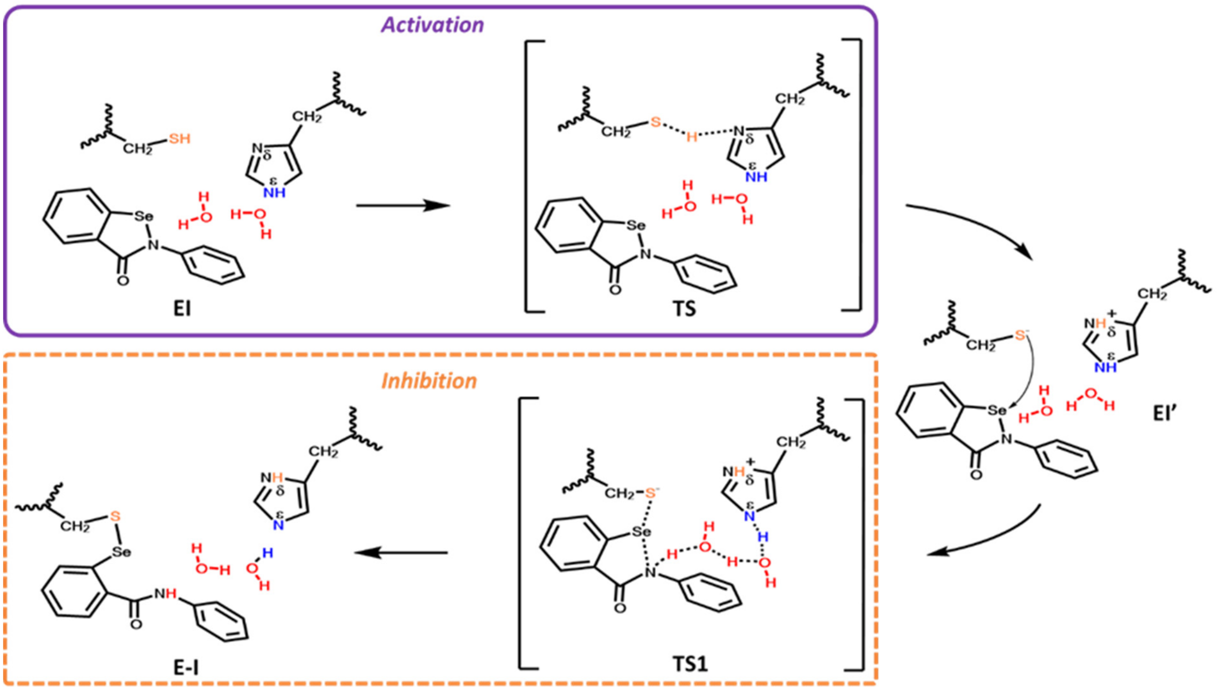


**Scheme S1.** *Proposed mechanism for the covalent inhibition of M^pro SARS-CoV-2 for HIE by EBS in two phases: activation (in violet) and inhibition through covalent bond formation (in dashed orange).*

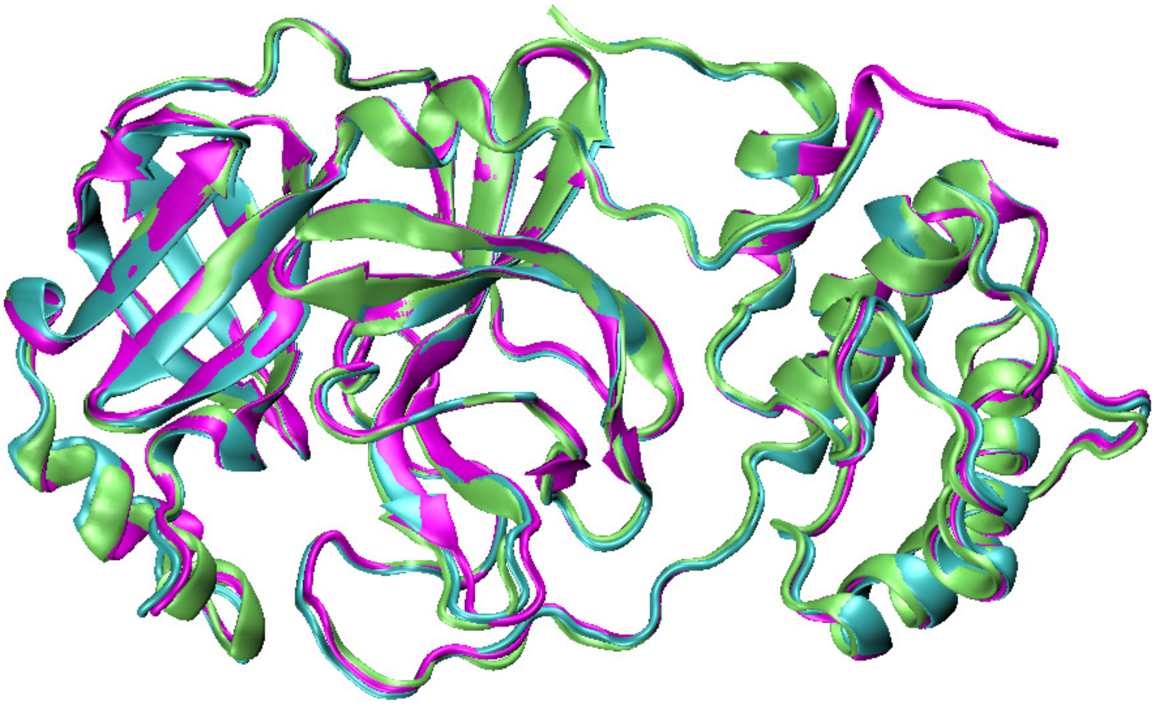

**Figure S1.** Superposition of M^pro crystallographic structures: 6W63 in cyano, 6LU7 in magenta, and 6Y2F in lime.

a)

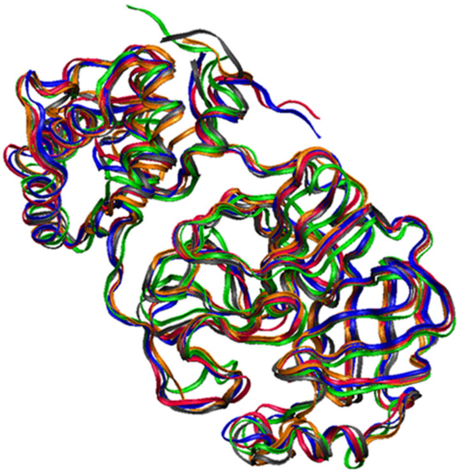

b)

| Structure | Color | RMSD |
|---|---|---|
| **Cluster 0** | Gray | 2.70 |
| **Cluser 1** | Orange | 2.78 |
| **Cluster 2** | Green | 3.59 |
| **Cluster 3** | Red | 1.19 |
| **Xray** | Blue | - |

a')

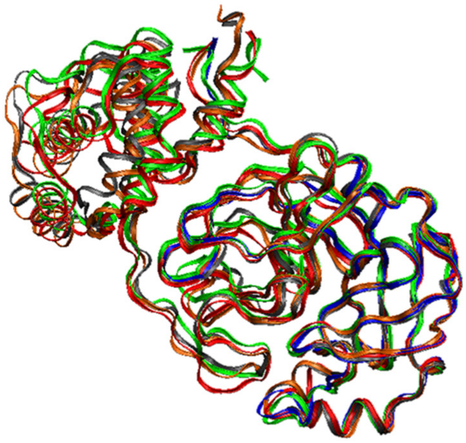

b')

| Structure | Color | RMSD |
|---|---|---|
| **Cluster 0** | Gray | 2.26 |
| **Cluser 1** | Orange | 2.11 |
| **Cluster 2** | Green | 1.77 |
| **Cluster 3** | Red | 1.74 |
| **Xray** | Blue | - |

**Figure S2.** Superposition of 4 most populated structures obtained by RMSD-based clustering MD simulation of (a) $M^{pro}$ HIE and (a') $M^{pro}$ HID. RMSD values of HIE clusters (b) with respect to X-Ray 6W63, RMSD values of HID clusters (b') with respect to X-Ray 6W63.

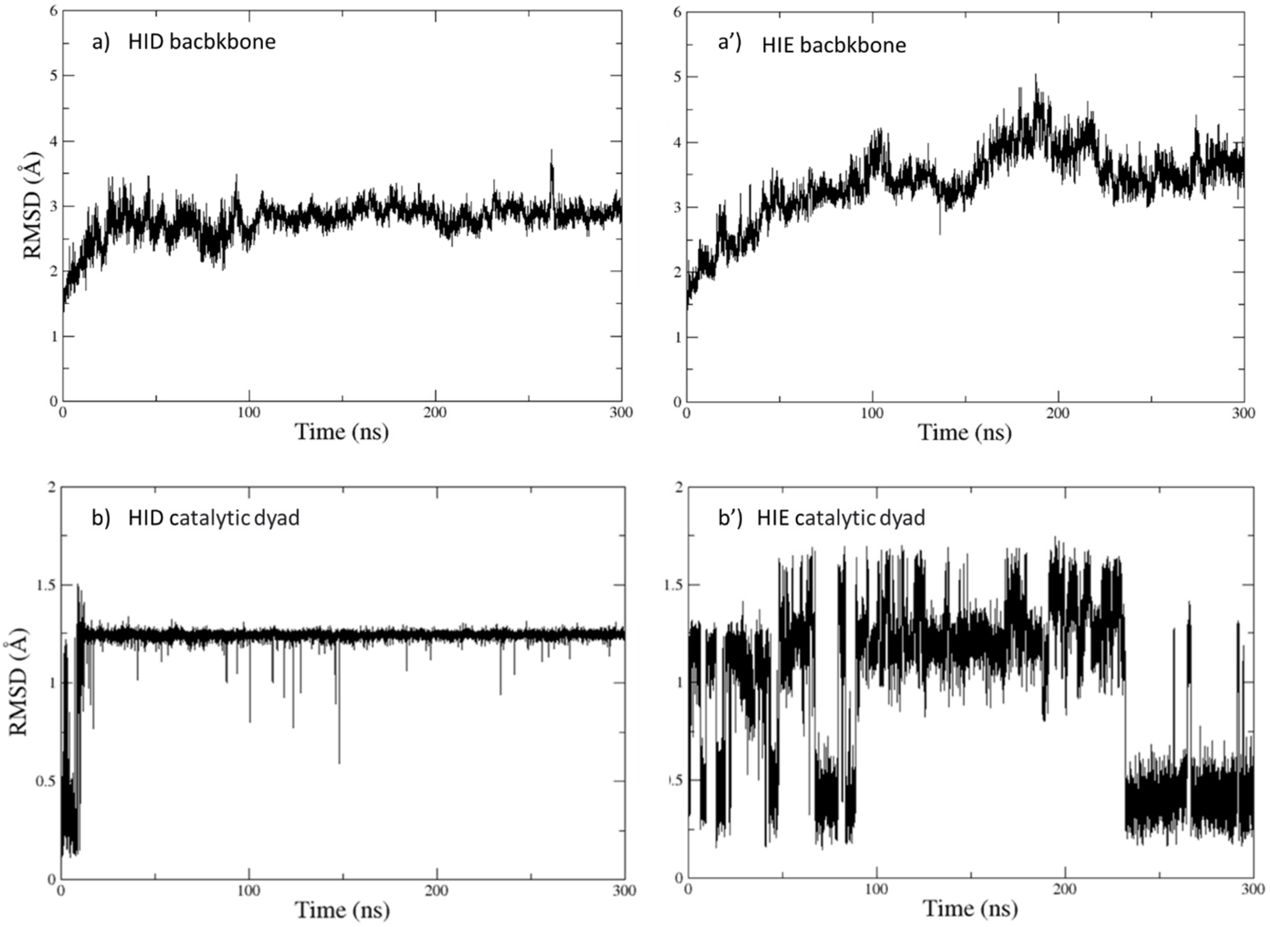


**Figure S3.** Up: backbone RMSD trend of a) M$^{pro}$ (HID) and a') M$^{pro}$ (HIE) along MD simulation time, expressed in Å. Down: RMSD trend of residues His41 and Cys145 b) M$^{pro}$ (HID) and b') M$^{pro}$ (HIE).

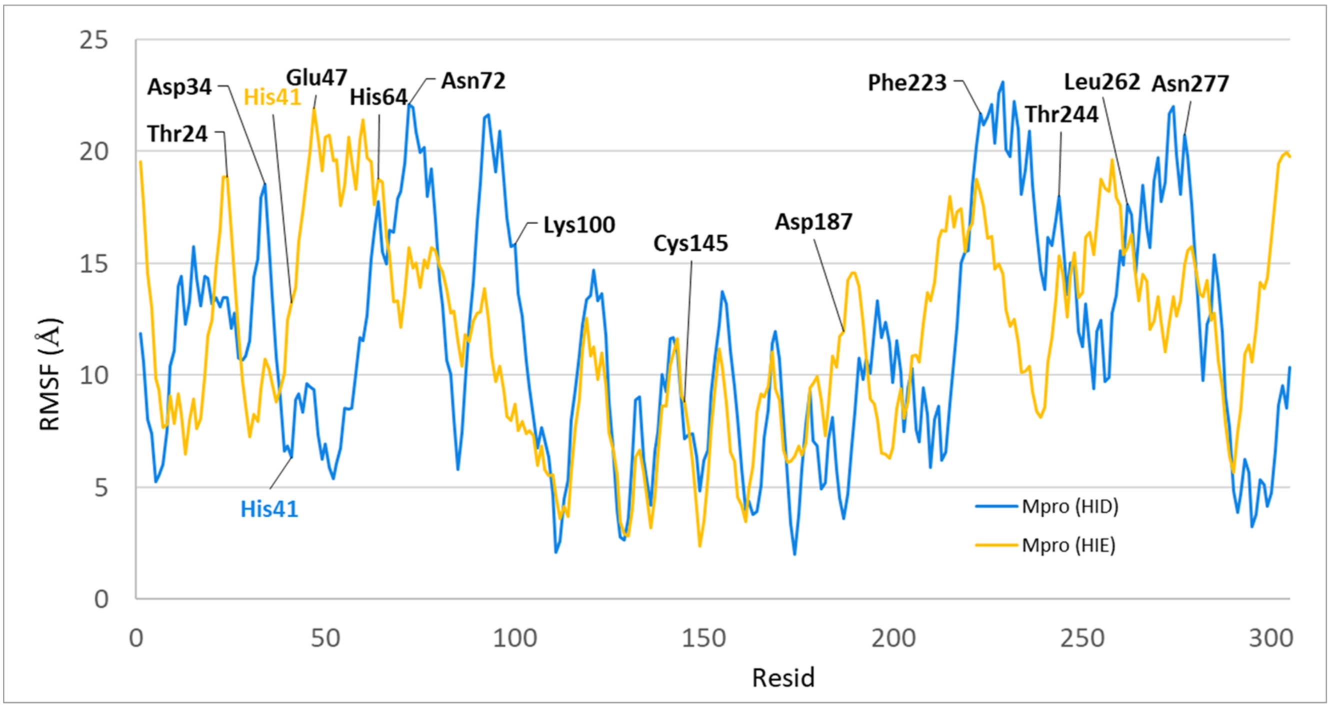


**Figure S4.** RMSF comparison between $M^{pro}$ (HID) in blue and $M^{pro}$ (HIE) in yellow; some residues with high DRMSF value between the two protonation states of His41 simulations are explicitly reported in the graph.

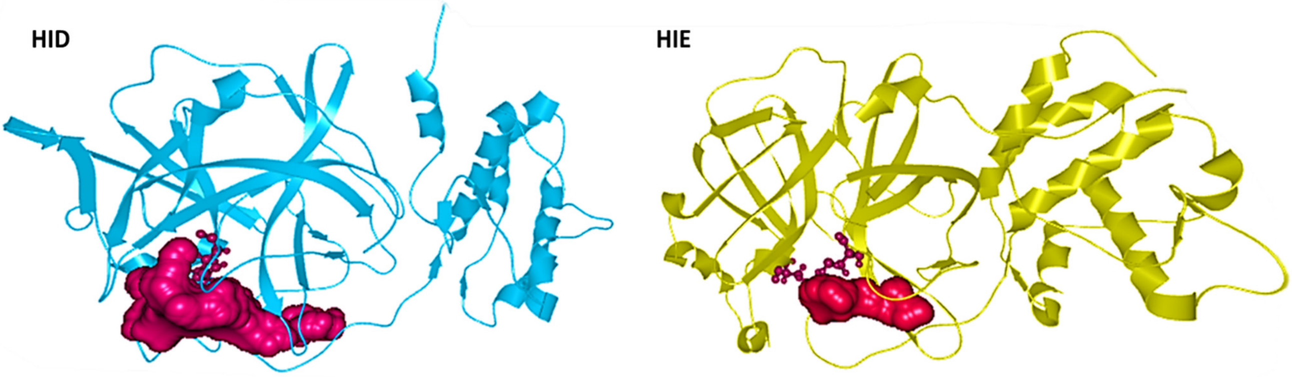


**Figure S5.** Pocket volume cavity of binding site: value of 598.1 Å$^3$ for $M^{pro}$ HID (in blue) and value of 227.7 Å3 $M^{pro}$ HIE (in gold). The calculation was performed with CAVER Analyst 2.0. 5 Å sphere size used to define the pocket of each system for volume calculations.

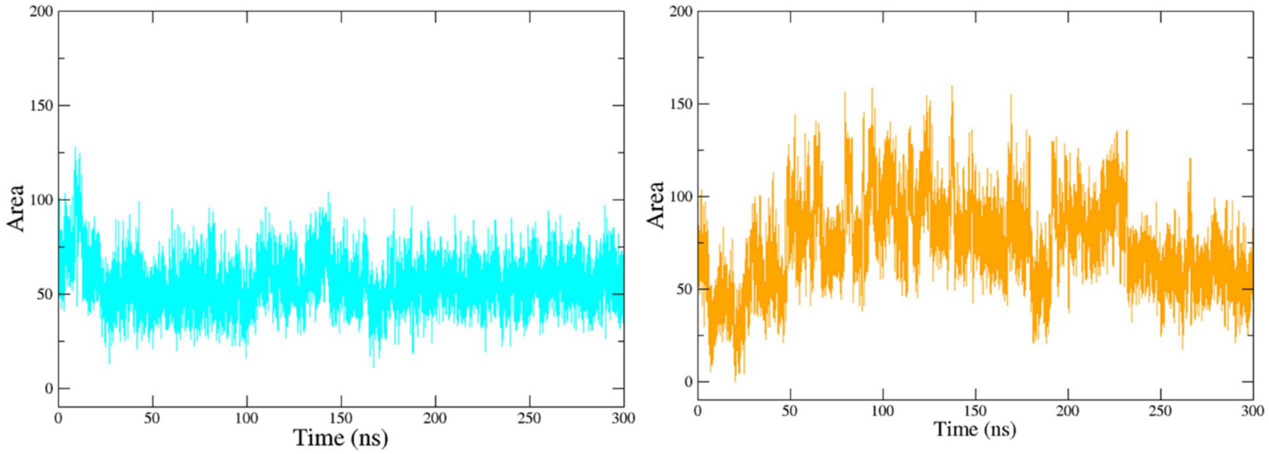


**Figure S6.** Solvent accessibility surface area (SASA) along the trajectory, expressed in Å$^2$ and calculated based on the residues Cys145 and His41 in different protonation states: HID in orange and HID in blue. SASA was calculated with CPPTRAJ using LCPO algorithm.[15]

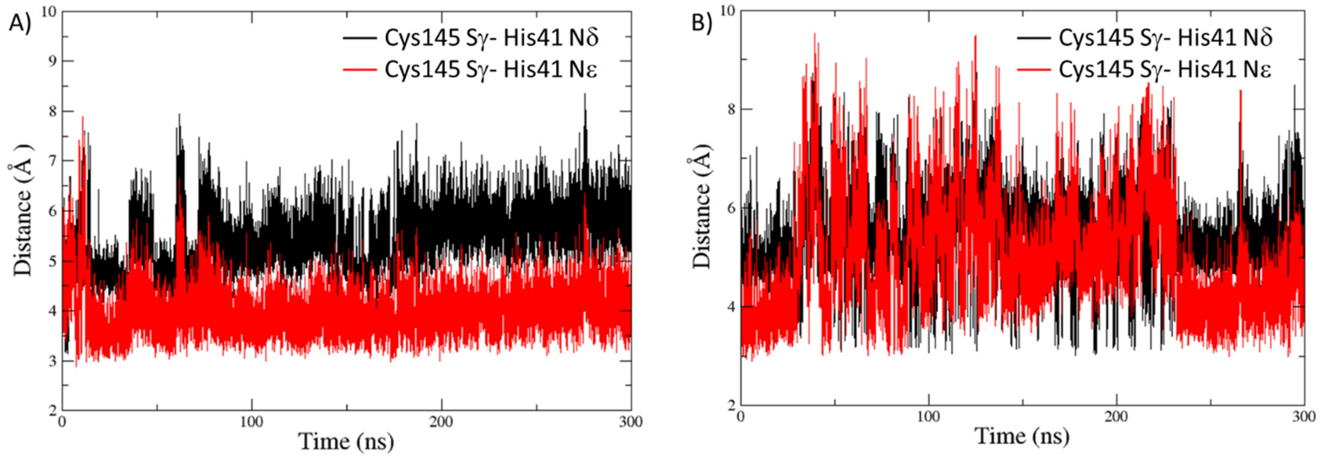


**Figure S7.** Distance between N and S (red) and N and S (black) in the catalytic residues His41 and Cys145. Graph A) refers to HID and graph B) refers to HIE trajectories.

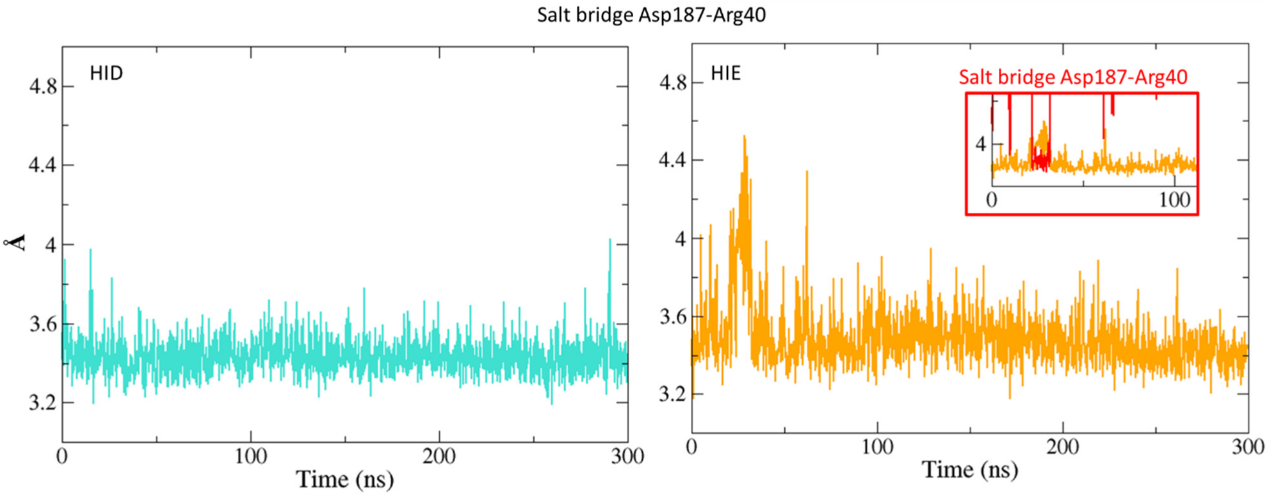


**Figure S8.**Salt bridges between residues Asp186 and Arg40 along the MD simulation trajectory, for HID and HIE, cyano and orange respectively. For the HIE trajectory, an additional data of salt bridge Asp187-Arg40 is zoom and shown in the red square. Analysis was performed by VMD plugin Version 1.1 with the cutoff distance of 3.2 Å.

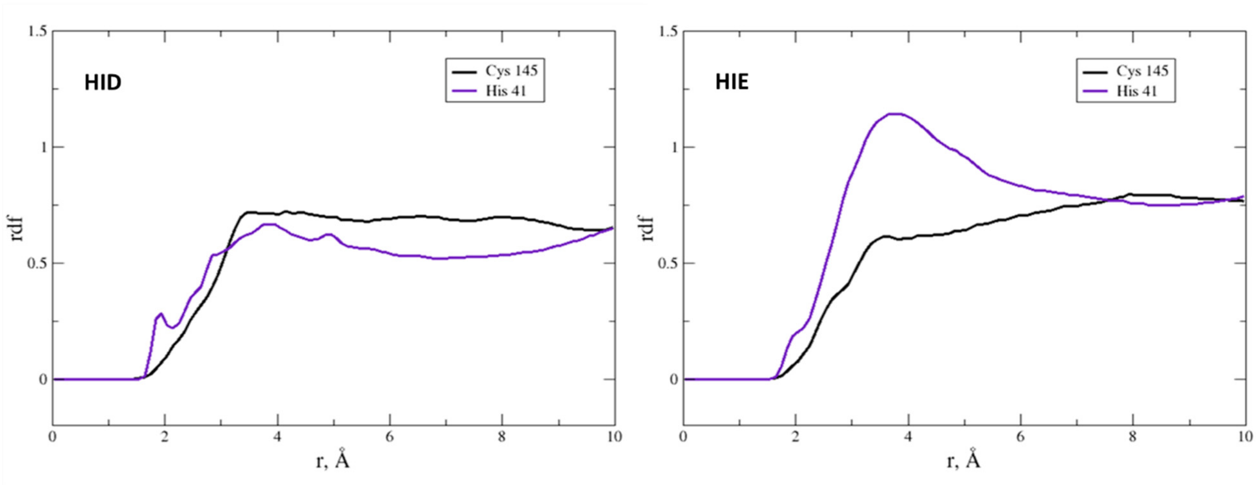


**Figure S9.** Water radial distribution function (RDF) analysis obtained as function of the distance (r) expressed in Å between water oxygen and side chains of Cys145 (black line) and His41 (purple line), for HID on left and for HIE on right.

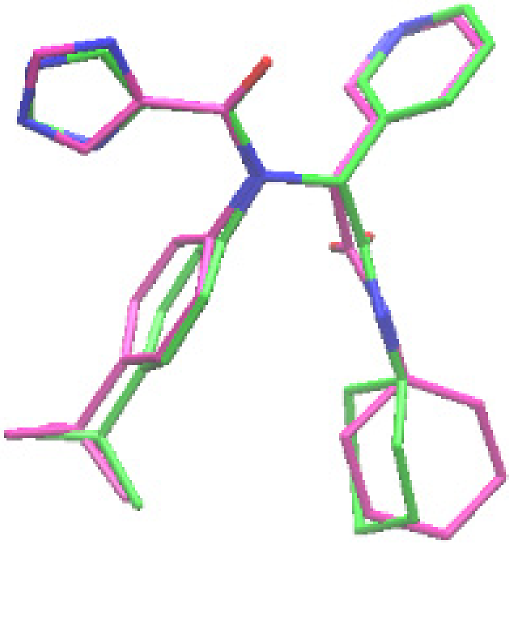

**Figure S10.** Superposition of **X77** structure obtained by the best docking pose (in pink) and X-Ray structure (colored by atom) with a RMSD of 1.91 Å.

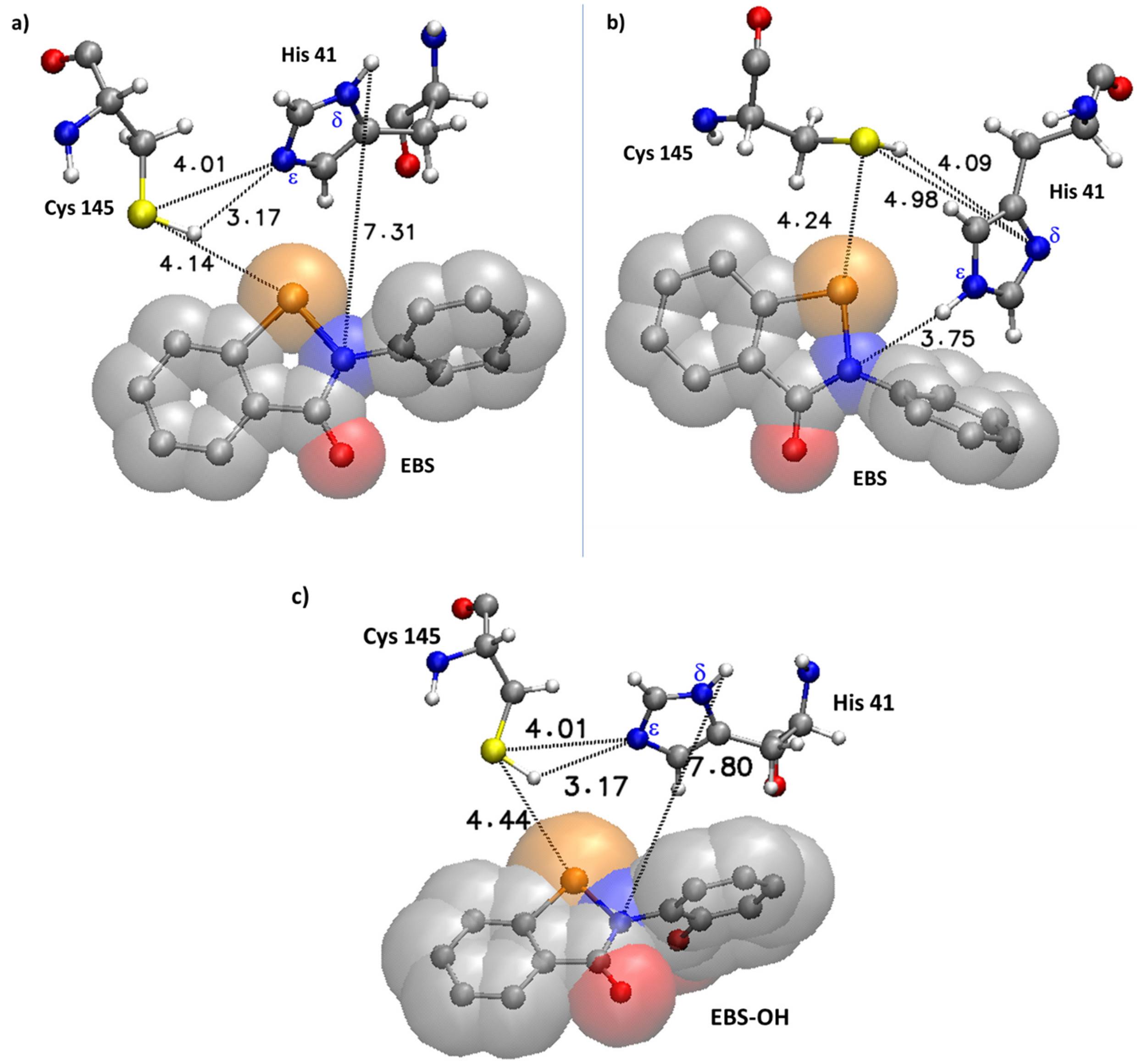


**Figure S11.** Structure and main distances (in Å) between atoms of M^pro^ active site and inhibitor best docked pose, a) refers to EBS docked in the M^pro^ HID most populeted cluster, b) refers to EBS docked in the M^pro^ HIE most populeted cluster and c) is the best docked pose of EBS-OH in the catalitic pocket of M^pro^ HID; van der Waals radii of EBS and EBS-OH atoms are shown in transparent.

| HID-EBS | EI | TS | EI′ | TS1 | E-I |
|---|---|---|---|---|---|
| *d* (O-ND2) Å | 2.94 | 3.13 | 2.99 | 3.10 | 2.78 |
| *d′* (O-HD22) Å | 1.95 | 2.25 | 2.12 | 2.22 | 1.75 |

| HIE-EBS | EI | TS | EI′ | TS1 | E-I |
|---|---|---|---|---|---|
| *d* (O-ND2) Å | 4.49 | 4.49 | 3.83 | 3.60 | 3.13 |
| *d′* (O-HD22) Å | 3.93 | 3.12 | 3.33 | 3.11 | 2.46 |

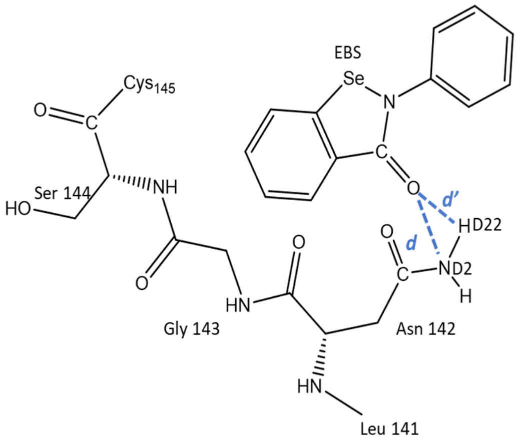


**Figure S12.** Tables containing distances between EBS oxygen atom and Asn 142 atoms ND2 (d) and HDD2 (d′) as depicted in the included scheme on the right. Table on top refers to HID; table down refers to HIE.

| HID-EBS-OH | EI | TS | EI′ | TS1 | E-I |
|---|---|---|---|---|---|
| *d* (O-ND2) Å | 5.95 | 3.04 | 3.02 | 2.83 | 2.73 |
| *d′* (O-HD22) Å | 5.12 | 2.66 | 2.42 | 1.83 | 1.74 |
| *d* (H-OG) Å | 1.77 | 1.67 | 1.68 | 1.88 | 1.81 |

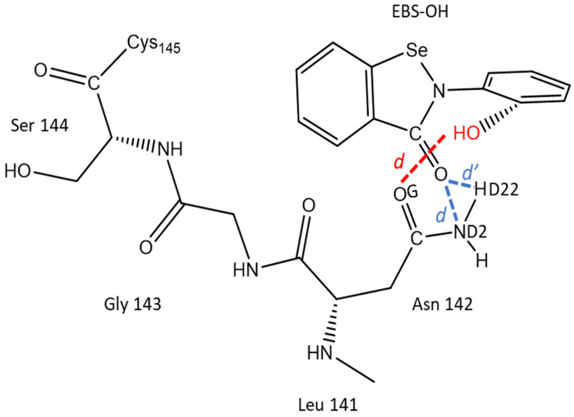


**Figure S13.** Tables containing distances between some selected atoms of EBS-OH and Asn 142 side chain. Distance between carbonyl oxygen O of EBS-OH and Asn 142 ND2 (d)/HDD2 (d′) are in blue, ditance between H of EBS-OH hydroxyl group and OG of Asn 142 is in red, as depicted in the included scheme on the right.

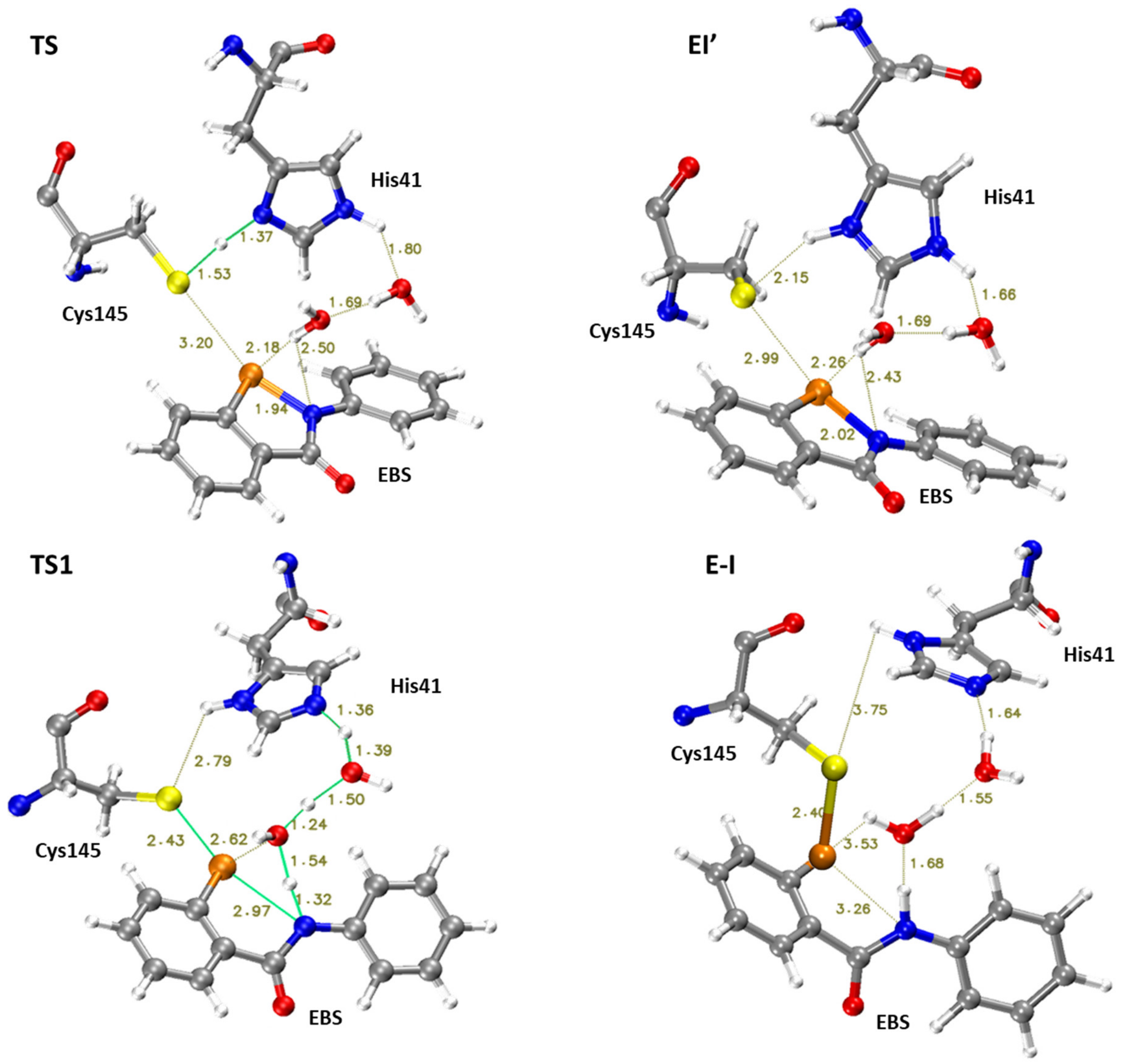


**Figure S14.** Optimized structures of the stationary points involved in the M[pro] HIE inhibition process promoted by EBS. For clarity, only residues of the model implicated in the chemical event are shown. Main distances are in Å. Imaginary frequencies are reported in $cm^{-1}$ for all transition states.

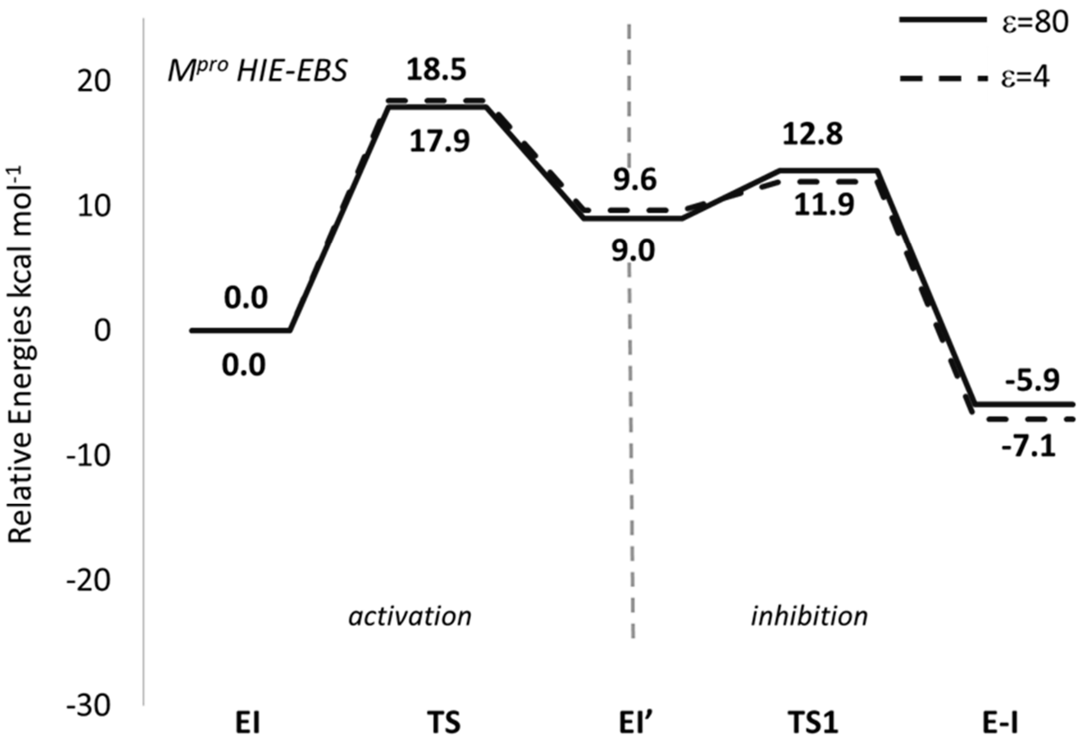


**Figure S15.** B3LYP-D3/6-31+G (2d,2p) free energy profiles of M$^{pro}$ HIE SARS-CoV-2 covalent inhibition mechanism by EBS in two phases: activation and inhibition through covalent bond formation, expressed in kcal mol-1. Solid line indicates calculations in water (Ɛ=80) and dotted line in protein environment (Ɛ=4).

**Table S1.** Calculated pKa for ionizable residues of M$^{pro}$. Residues fully protonated (positively charged) and deprotonated (negatively charged) are colored in blue and red respectively. Residues belonging to the catalytic dyad are in bold.

| | ***pKa*** | **Residue** | ***pKa*** |
|---|---|---|---|
| Arg4 | 11.81 | Asp153 | 4.40 |
| Lys5 | 8.45 | Tyr154 | 9.67 |
| Lys12 | 9.30 | Asp155 | 4.37 |
| Glu14 | 3.81 | Cys156 | 9.62 |
| Cys16 | 12.15 | Cys160 | 10.25 |
| Cys22 | 11.90 | Tyr161 | 11.64 |
| Asp33 | 3.87 | His163 | 4.17 |
| Asp34 | 4.50 | His164 | 3.75 |
| Tyr37 | 11.32 | Glu166 | 4.67 |
| Cys38 | 12.12 | His172 | 4.08 |
| Arg40 | 11.20 | Asp176 | 2.86 |
| **His41** | **4.42** | Glu178 | 3.92 |
| Cys44 | 9.99 | Tyr182 | 13.32 |
| Glu47 | 4.10 | Asp187 | 5.09 |
| Asp48 | 3.37 | Arg188 | 11.61 |
| Tyr54 | 13.17 | Asp197 | 4.16 |
| Glu55 | 4.55 | Tyr209 | 13.34 |
| Asp56 | 4.46 | Asp216 | 4.29 |
| Arg60 | 12.16 | Arg217 | 11.81 |
| Lys61 | 10.19 | Arg222 | 11.97 |
| His64 | 6.37 | Asp229 | 4.15 |
| Arg76 | 11.77 | Lys236 | 10.40 |
| His80 | 7.39 | Tyr237 | 10.96 |
| Cys85 | 10.18 | Tyr239 | 11.16 |
| Lys88 | 10.65 | Glu240 | 4.60 |
| Lys90 | 10.18 | Asp245 | 3.56 |
| Asp92 | 3.81 | His246 | 3.97 |
| Lys97 | 10.43 | Asp248 | 4.05 |
| Lys100 | 10.04 | Asp263 | 3.92 |
| Tyr101 | 10.51 | Cys265 | 11.78 |
| Lys102 | 9.91 | Lys269 | 9.18 |
| Arg105 | 11.70 | Glu270 | 4.58 |
| Cys117 | 10.91 | Arg279 | 12.06 |
| Tyr118 | 11.68 | Glu288 | 4.07 |
| Tyr126 | 13.65 | Asp289 | 3.38 |
| Cys128 | 10.29 | Glu290 | 4.52 |
| Arg131 | 10.66 | Asp295 | 4.14 |
| Lys137 | 9.56 | Arg298 | 10.30 |
| **Cys145** | **8.94** | Cys300 | 10.05 |

**Table S2.** Donor-Acceptor hydrogen bond details of 300 ns $M^{pro}$ HID simulation. Only H-bonds involving the side chains atoms of amino acid residues have been reported It also shows the occupancy of each hydrogen bond.

| A residue | A atom | D residue | D atom | Freq |
|---|---|---|---|---|
| GLU240 | O | THR201 | OG1 | 0 .9789 |
| ASN133 | OD1 | THR135 | OG1 | 0 .9790 |
| CYS160 | O | TYR182 | OH | 0 .9791 |
| ASP176 | O | THR175 | OG1 | 0 .9793 |
| ASP187 | OD1 | ARG40 | NH2 | 0 .9794 |
| ASP263 | O | SER267 | OG | 0 .9797 |
| **ASP187** | **OD2** | **ARG40** | **NE** | **0 .9798** |
| ASN28 | OD1 | GLY120 | N | 0 .9804 |
| ILE259 | O | SER254 | OG | 0 .9807 |
| ILE259 | O | TYR209 | OH | 0 .9808 |
| PHE181 | O | ARG105 | NH2 | 0 .9811 |
| LEU253 | O | THR257 | OG1 | 0 .9812 |
| LEU141 | O | SER144 | OG | 0 .9815 |
| GLN127 | OE1 | SER113 | OG | 0 .9819 |
| ASP289 | O | ASN203 | ND2 | 0 .9821 |
| TRP31 | O | ASN95 | ND2 | 0 .9824 |
| GLY195 | O | ASN133 | ND2 | 0 .9836 |
| ASN231 | OD1 | LEU242 | N | 0 .9838 |
| CYS145 | O | ASN28 | ND2 | 0 .9841 |
| ASP289 | OD2 | ARG131 | NH2 | 0 .9844 |
| ASP295 | OD2 | THR111 | OG1 | 0 .9852 |
| ASN180 | O | ARG105 | NH1 | 0 .9863 |
| ASN65 | OD1 | SER62 | N | 0 .9868 |
| SER113 | OG | PHE8 | N | 0 .9870 |
| ASN95 | OD1 | LYS97 | N | 0 .9878 |
| THR280 | O | TRP218 | NE1 | 0 .9883 |
| ASP176 | OD2 | ARG105 | NH1 | 0 .9885 |
| ALA210 | O | ASN214 | ND2 | 0 .9886 |
| LEU286 | O | SER284 | OG | 0 .9887 |

**Table S3.** Donor-Acceptor hydrogen bond details of 300 ns $M^{pro}$ HIE simulation. Only H-bonds involving the side chains atoms of amino acid residues have been reported. It also shows the occupancy of each hydrogen bond.

| A residue | A atom | D residue | D atom | Freq |
|---|---|---|---|---|
| CYS160 | O | TYR182 | OH | 0.9519 |
| GLU240 | O | THR201 | OG1 | 0.9502 |
| ASP263 | O | SER267 | OG | 0.8845 |
| ASN133 | OD1 | THR135 | OG1 | 0.8843 |
| ASP176 | O | THR175 | OG1 | 0.8626 |
| ASP187 | OD1 | ARG40 | NH2 | 0.8189 |
| ILE259 | O | TYR209 | OH | 0.7847 |
| ASN28 | OD1 | GLY120 | N | 0.7741 |
| ILE259 | O | SER254 | OG | 0.7659 |
| LEU253 | O | THR257 | OG1 | 0.7652 |
| LEU141 | O | SER144 | OG | 0.7566 |
| ASN231 | OD1 | LEU242 | N | 0.7233 |
| TRP31 | O | ASN95 | ND2 | 0.7071 |
| THR175 | O | MET162 | N | 0.7016 |
| ASP187 | OD2 | ARG40 | NE | 0.6625 |
| ASP289 | O | ASN203 | ND2 | 0.6545 |
| PHE181 | O | ARG105 | NH2 | 0.6023 |
| CYS145 | O | ASN28 | ND2 | 0.5805 |
| SER144 | O | SER147 | OG | 0.5753 |
| ASP48 | OD2 | THR45 | OG1 | 0.5590 |
| LEU286 | O | SER284 | OG | 0.5453 |
| ASN95 | OD1 | LYS97 | N | 0.5163 |
| ASP176 | OD1 | ASN180 | N | 0.5045 |

**Table S4.** Tables of 10 structures obtained by RMSD-based clustering of MD simulations. $M^{pro}$ HID (top) and $M^{pro}$ HIE (down). From first column: cluster name, number of frames included in the cluster, population respect to total processed frames in percentage, average distance between frames inside the cluster expressed in Å, standard deviation related to Avg d and the centroid frame.

| Name | Frames | Pop % | Avg d (Å) | σ | Centroid |
|---|---|---|---|---|---|
| **Cluster 0** | 6594 | 44.0 | 2.09 | 0.40 | 9055 |
| **Cluster 1** | 3467 | 23.1 | 2.06 | 0.41 | 10699 |
| **Cluster 2** | 1978 | 13.2 | 2.01 | 0.38 | 1017 |
| **Cluster 3** | 999 | 6.7 | 1.86 | 0.36 | 2320 |
| **Cluster 4** | 699 | 4.7 | 2.00 | 0.39 | 227 |
| **Cluster 5** | 675 | 4.5 | 1.90 | 0.39 | 469 |
| **Cluster 6** | 269 | 1.8 | 1.72 | 0.43 | 4677 |
| **Cluster 7** | 176 | 1.2 | 1.64 | 0.38 | 64 |
| **Cluster 8** | 108 | 0.7 | 1.37 | 0.19 | 13153 |
| **Cluster 9** | 35 | 0.2 | 0.00 | 0.00 | 16 |

| Name | Frames | Pop % | Avg d (Å) | σ | Centroid |
|---|---|---|---|---|---|
| **Cluster 0** | 5012 | 25.1 | 1.71 | 0.34 | 15341 |
| **Cluster 1** | 4213 | 21.1 | 1.86 | 0.39 | 7693 |
| **Cluster 2** | 3456 | 17.3 | 1.74 | 0.38 | 18467 |
| **Cluster 3** | 2302 | 11.5 | 1.60 | 0.33 | 939 |
| **Cluster 4** | 1553 | 7.8 | 1.66 | 0.37 | 9760 |
| **Cluster 5** | 1551 | 7.8 | 1.60 | 0.29 | 2581 |
| **Cluster 6** | 1276 | 6.4 | 1.50 | 0.25 | 4517 |
| **Cluster 7** | 323 | 1.6 | 1.38 | 0.26 | 3995 |
| **Cluster 8** | 290 | 1.5 | 1.38 | 0.30 | 17856 |
| **Cluster 9** | 25 | 0.1 | 0.00 | 0.00 | 2349 |

**Table S5.** Docking scores values related to the free energy of protein-ligand binding affinity expressed in kcal mol$^{-1}$ and it's relative RMSD in Å. a) $M^{pro}$ HID most representative cluster and EBS, b) $M^{pro}$ HIE most representative cluster and EBS and c) $M^{pro}$ HID with EBS-OH. Results are reported for all 10 docking poses ranked by energy. Best docking poses values are shown in red.

| | **a) $M^{pro}$ HID and EBS** | | **b) $M^{pro}$ HIE and EBS** | | **c) $M^{pro}$ HID and EBS-OH** | |
|---|---|---|---|---|---|---|
| Pose | E (kcal mol$^{-1}$) | RMSD (Å) | E (kcal mol$^{-1}$) | RMSD (Å) | E (kcal mol$^{-1}$) | RMSD (Å) |
| 1 | -5.85 | 0.00 | -6.39 | 0.00 | -5.67 | 0.00 |
| 2 | -5.85 | 0.02 | -6.39 | 0.01 | -5.48 | 1.59 |
| 3 | -5.85 | 0.01 | -6.39 | 0.02 | -5.47 | 1.58 |
| 4 | -5.85 | 0.06 | -6.39 | 0.02 | -5.27 | 1.75 |
| 5 | -5.85 | 0.05 | -6.38 | 0.11 | -5.27 | 1.74 |
| 6 | -5.85 | 0.05 | -6.38 | 0.14 | -5.26 | 1.73 |
| 7 | -5.84 | 0.08 | -6.38 | 0.12 | -5.23 | 0.45 |
| 8 | -5.84 | 0.07 | -6.38 | 0.06 | -5.21 | 1.71 |
| 9 | -5.84 | 0.11 | -6.38 | 0.09 | -5.18 | 1.39 |
| 10 | -5.55 | 0.00 | -6.38 | 0.04 | -5.04 | 1.37 |

**Table S6.** NBO charge values of heavy atoms in |e| for EBS and EBS-OH. Atoms numbering follows the sketch on the right.

| | EBS | EBS-OH |
|---|---|---|
| Se 1 | 0.634 | 0.622 |
| N 2 | -0.602 | -0.661 |
| C 3 | 0.673 | 0.665 |
| O 3 | -0.618 | -0.597 |
| C 4 | -0.177 | -0.177 |
| C 5 | -0.252 | -0.252 |
| C 6 | -0.213 | -0.212 |
| C 7 | -0.257 | -0.258 |
| C 1' | 0.152 | 0.053 |
| C 2' | -0.254 | -0.202 |
| C 3' | -0.229 | -0.270 |
| C 4' | -0.250 | -0.218 |
| C 5' | -0.231 | -0.285 |
| C 6' | -0.273 | 0.322 |
| O 6' (OH) | - | -0.702 |

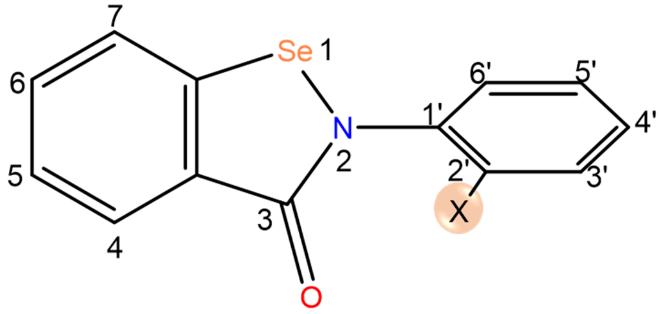

**Table S7.** NBO charge values of heavy atoms in |e| for every stationary point along the potential energy surface of inhibition promoted by EBS on M^pro HID. Heavy atoms of the inhibitor, of the two catalytic waters, of the catalytic dyad and of the residues immediately close to the dyad are reported.

| Residue | Atom | EI | TS | EI' | TS1 | E-I |
|---|---|---|---|---|---|---|
| His 41 | N | -0.100 | -0.027 | -0.002 | -0.224 | -0.066 |
| His 41 | CA | 0.591 | 0.123 | -0.015 | 0.181 | 0.337 |
| His 41 | CB | -0.912 | -0.857 | -0.727 | 0.124 | -0.885 |
| His 41 | CG | 0.515 | 0.599 | 0.375 | 0.145 | -0.079 |
| His 41 | ND1 | -0.219 | -0.003 | -0.007 | 0.152 | -0.193 |
| His 41 | CE1 | 0.469 | 0.135 | 0.908 | 0.315 | 0.266 |
| His 41 | NE2 | -0.223 | 0.274 | -0.026 | 0.066 | 0.086 |
| His 41 | CD2 | 0.069 | 0.189 | 0.374 | 0.139 | 0.639 |
| His 41 | C | 0.163 | 0.410 | 0.464 | 0.597 | 0.236 |
| His 41 | O | -0.375 | -0.424 | -0.427 | -0.598 | -0.364 |
| Asn 142 | N | 0.044 | -0.088 | -0.097 | -0.181 | 0.140 |
| Asn 142 | CA | 0.068 | 0.683 | 0.691 | 0.252 | -0.109 |
| Asn 142 | CB | -0.231 | -0.312 | -0.326 | -0.025 | -0.273 |
| Asn 142 | CG | 0.813 | 0.550 | 0.541 | 0.949 | 0.853 |
| Asn 142 | OD1 | -0.472 | -0.454 | -0.449 | -0.788 | -0.464 |
| Asn 142 | ND2 | 0.029 | 0.126 | 0.123 | -0.180 | 0.134 |
| Asn 142 | C | 0.073 | -0.306 | -0.290 | 0.791 | -0.081 |
| Asn 142 | O | -0.475 | -0.510 | -0.516 | -0.752 | -0.435 |
| Gly 143 | N | 0.259 | 0.170 | 0.174 | -0.313 | 0.129 |
| Gly 143 | CA | -0.171 | 0.075 | 0.074 | 0.296 | -0.267 |
| Gly 143 | C | 0.345 | 0.181 | 0.173 | 0.836 | 0.680 |
| Gly 143 | O | -0.338 | -0.402 | -0.405 | -0.704 | -0.492 |
| Ser 144 | N | 0.217 | 0.215 | 0.226 | -0.405 | 0.311 |
| Ser 144 | CA | 0.113 | 0.364 | 0.384 | 0.240 | 0.343 |
| Ser 144 | CB | -0.039 | 0.123 | 0.115 | 0.387 | -0.174 |
| Ser 144 | OG | -0.107 | -0.098 | -0.099 | -0.298 | -0.093 |
| Ser 144 | C | 0.479 | 0.141 | 0.115 | 1.043 | 0.178 |
| Ser 144 | O | -0.533 | -0.572 | -0.585 | -0.925 | -0.511 |
| Cys 145 | N | 0.298 | 0.373 | 0.344 | -0.509 | 0.407 |
| Cys 145 | CA | 0.683 | -0.135 | -0.290 | 0.196 | 0.530 |
| Cys 145 | CB | -0.782 | -0.528 | -0.500 | 0.061 | -0.495 |
| Cys 145 | SG | 0.143 | -0.074 | 0.542 | -0.461 | -0.077 |
| Cys 145 | C | -0.092 | 0.129 | 0.439 | 1.020 | -0.447 |
| Cys 145 | O | -0.414 | -0.387 | -0.527 | -0.960 | -0.389 |
| Gly 146 | N | 0.086 | 0.068 | 0.074 | -0.473 | 0.099 |
| Gly 146 | CA | 0.163 | 0.189 | 0.178 | 0.260 | 0.149 |
| Gly 146 | C | 0.471 | 0.477 | 0.475 | 0.694 | 0.498 |
| Gly 146 | O | -0.415 | -0.402 | -0.407 | -0.653 | -0.397 |
| EBS | C5 | -0.154 | -0.109 | -0.119 | -0.038 | 0.217 |
| EBS | C6 | 0.257 | -0.194 | -0.238 | 0.058 | -0.017 |
| EBS | C4 | -0.224 | -0.518 | -0.520 | 0.083 | -0.742 |
| EBS | C7 | 0.353 | 0.737 | 0.806 | -0.128 | 0.981 |

| | | | | | | |
|---|---|---|---|---|---|---|
| EBS | C(-CO) | -0.797 | 0.068 | 0.028 | -0.196 | 0.287 |
| EBS | C(-Se) | 0.586 | -0.248 | -0.287 | 0.153 | -0.761 |
| EBS | C3 | 0.004 | -0.354 | -0.346 | 0.929 | 1.042 |
| EBS | N2 | -0.265 | -0.002 | 0.011 | -0.378 | 0.162 |
| EBS | O | -0.460 | -0.530 | -0.546 | -0.860 | -0.506 |
| EBS | Se1 | 0.343 | 0.614 | 0.714 | 0.666 | -0.258 |
| EBS | C1’ | -1.152 | -0.077 | -0.129 | 0.332 | -0.748 |
| EBS | C6’ | 1.461 | 0.307 | 0.329 | -0.048 | -0.230 |
| EBS | C5’ | -0.946 | -0.416 | -0.434 | -0.004 | 0.279 |
| EBS | C4’ | 0.420 | 0.176 | 0.160 | -0.053 | 0.211 |
| EBS | C3’ | -0.525 | 0.052 | 0.052 | 0.011 | -0.208 |
| EBS | C2’ | 0.750 | 0.344 | 0.326 | -0.033 | 0.550 |
| W1 | O | 0.071 | 0.085 | 0.082 | -0.846 | -0.053 |
| W2 | O | 0.085 | 0.053 | 0.103 | 0.058 | 0.070 |

**Table S8.** NBO charge values of heavy atoms in |e| for every stationary point along the potential energy surface of inhibition promoted by EBS on $M^{pro}$ HIE. Heavy atoms of the inhibitor, of the two catalytic waters, of the catalytic dyad and of the residues immediately close to the dyad are reported.

| Residue | Atom | EI | TS | EI' | TS1 | E-I |
|---|---|---|---|---|---|---|
| His 41 | N | -0.609 | -0.576 | -0.583 | -0.585 | -0.584 |
| His 41 | CA | 0.003 | -0.029 | -0.005 | -0.005 | -0.014 |
| His 41 | CB | -0.310 | -0.327 | -0.332 | -0.333 | -0.327 |
| His 41 | CG | 0.128 | 0.182 | 0.080 | 0.302 | 0.266 |
| His 41 | ND1 | -0.570 | -0.580 | -0.430 | -0.697 | -0.720 |
| His 41 | CE1 | 0.228 | 0.342 | 0.232 | 0.369 | 0.365 |
| His 41 | NE2 | -0.704 | -0.666 | -0.667 | -0.612 | -0.578 |
| His 41 | CD2 | 0.087 | 0.095 | 0.111 | 0.016 | 0.002 |
| His 41 | C | 0.377 | 0.388 | 0.352 | 0.347 | 0.381 |
| His 41 | O | -0.421 | -0.425 | -0.418 | -0.403 | -0.418 |
| Asn 142 | N | -0.538 | -0.539 | -0.536 | -0.527 | -0.537 |
| Asn 142 | CA | -0.040 | -0.040 | -0.050 | -0.060 | -0.038 |
| Asn 142 | CB | -0.317 | -0.317 | -0.312 | -0.323 | -0.328 |
| Asn 142 | CG | 0.547 | 0.551 | 0.539 | 0.559 | 0.575 |
| Asn 142 | OD1 | -0.508 | -0.511 | -0.498 | -0.508 | -0.536 |
| Asn 142 | ND2 | -0.719 | -0.718 | -0.716 | -0.715 | -0.681 |
| Asn 142 | C | 0.521 | 0.497 | 0.513 | 0.524 | 0.507 |
| Asn 142 | O | -0.503 | -0.486 | -0.491 | -0.494 | -0.493 |
| Gly 143 | N | -0.655 | -0.656 | -0.666 | -0.648 | -0.645 |
| Gly 143 | CA | -0.126 | -0.176 | -0.182 | -0.166 | -0.171 |
| Gly 143 | C | 0.546 | 0.553 | 0.509 | 0.535 | 0.540 |
| Gly 143 | O | -0.497 | -0.511 | -0.456 | -0.518 | -0.521 |
| Ser 144 | N | -0.604 | -0.608 | -0.640 | -0.616 | -0.605 |
| Ser 144 | CA | -0.092 | -0.052 | -0.080 | -0.066 | -0.058 |
| Ser 144 | CB | -0.031 | -0.042 | -0.024 | -0.034 | -0.041 |
| Ser 144 | OG | -0.619 | -0.624 | -0.635 | -0.616 | -0.620 |
| Ser 144 | C | 0.602 | 0.579 | 0.603 | 0.548 | 0.570 |
| Ser 144 | O | -0.567 | -0.550 | -0.495 | -0.495 | -0.539 |
| Cys 145 | N | -0.645 | -0.622 | -0.616 | -0.641 | -0.634 |
| Cys 145 | CA | -0.034 | -0.030 | -0.019 | -0.043 | -0.016 |
| Cys 145 | CB | -0.494 | -0.473 | -0.501 | -0.486 | -0.517 |
| Cys 145 | SG | 0.009 | -0.169 | -0.430 | -0.115 | -0.110 |
| Cys 145 | C | 0.546 | 0.555 | 0.551 | 0.551 | 0.557 |
| Cys 145 | O | -0.472 | -0.487 | -0.466 | -0.484 | -0.473 |
| Gly 146 | N | -0.654 | -0.682 | -0.633 | -0.641 | -0.683 |
| Gly 146 | CA | -0.178 | -0.136 | -0.182 | -0.159 | -0.140 |
| Gly 146 | C | 0.360 | 0.346 | 0.368 | 0.352 | 0.351 |
| Gly 146 | O | -0.409 | -0.411 | -0.409 | -0.394 | -0.402 |
| EBS | C5 | -0.145 | -0.142 | -0.136 | -0.132 | -0.152 |
| EBS | C6 | -0.104 | -0.111 | -0.115 | -0.112 | -0.097 |
| EBS | C4 | -0.109 | -0.117 | -0.114 | -0.120 | -0.090 |
| EBS | C7 | -0.169 | -0.146 | -0.137 | -0.157 | -0.149 |

| | | | | | | |
|---|---|---|---|---|---|---|
| EBS | C(-CO) | 0.115 | 0.127 | 0.093 | 0.039 | 0.047 |
| EBS | C(-Se) | -0.381 | -0.384 | -0.395 | -0.337 | -0.248 |
| EBS | C3 | 0.540 | 0.540 | 0.498 | 0.494 | 0.490 |
| EBS | N2 | -0.944 | -0.924 | -0.952 | -0.789 | -0.826 |
| EBS | O | -0.467 | -0.470 | -0.430 | -0.501 | -0.424 |
| EBS | Se1 | 0.705 | 0.633 | 0.619 | 0.389 | 0.321 |
| EBS | C1’ | 0.334 | 0.341 | 0.286 | 0.304 | 0.458 |
| EBS | C6’ | -0.203 | -0.193 | -0.098 | -0.167 | -0.166 |
| EBS | C5’ | -0.158 | -0.146 | -0.137 | -0.145 | -0.141 |
| EBS | C4’ | -0.120 | -0.116 | -0.101 | -0.114 | -0.117 |
| EBS | C3’ | -0.169 | -0.167 | -0.147 | -0.155 | -0.115 |
| EBS | C2’ | -0.125 | -0.120 | -0.092 | -0.099 | -0.261 |
| W1 | O | -0.796 | -0.811 | -0.779 | -0.818 | -0.825 |
| W2 | O | -0.752 | -0.759 | -0.768 | -0.769 | -0.725 |

**Table S9.** NBO charge values of heavy atoms in |e| for every stationary point along the potential energy surface of inhibition promoted by EBS-OH on $M^{pro}$ HID. Heavy atoms of the inhibitor, of the two catalytic waters, of the catalytic dyad and of the residues immediately close to the dyad are reported.

| Residue | Atom | EI | TS | EI' | TS1 | E-I |
|---|---|---|---|---|---|---|
| His 41 | N | -0.046 | 0.053 | 0.053 | -0.006 | 0.009 |
| His 41 | CA | 0.360 | -0.235 | -0.007 | -0.034 | -0.009 |
| His 41 | CB | -0.796 | -0.308 | -0.942 | -0.586 | -0.141 |
| His 41 | CG | 0.150 | 0.823 | 0.513 | 0.360 | 0.333 |
| His 41 | ND1 | 0.214 | 0.035 | 0.133 | 0.211 | -0.044 |
| His 41 | CE1 | 0.129 | 0.146 | 0.374 | 0.046 | -0.183 |
| His 41 | NE2 | -0.212 | -0.363 | 0.141 | 0.215 | -0.217 |
| His 41 | CD2 | 0.209 | -0.255 | 0.771 | 0.615 | 0.416 |
| His 41 | C | 0.414 | 0.557 | 0.226 | 0.433 | 0.224 |
| His 41 | O | -0.343 | -0.401 | -0.368 | -0.403 | -0.369 |
| Asn 142 | N | 0.114 | 0.090 | 0.109 | -0.013 | 0.131 |
| Asn 142 | CA | -0.185 | 0.076 | -0.237 | 0.191 | -0.237 |
| Asn 142 | CB | -0.624 | -0.137 | -0.504 | -0.812 | -0.558 |
| Asn 142 | CG | 1.218 | 0.628 | 1.019 | 1.304 | 1.119 |
| Asn 142 | OD1 | -0.533 | -0.514 | -0.474 | -0.456 | -0.511 |
| Asn 142 | ND2 | 0.060 | 0.088 | 0.044 | 0.120 | 0.062 |
| Asn 142 | C | 0.180 | 0.029 | 0.079 | -0.036 | 0.433 |
| Asn 142 | O | -0.431 | -0.441 | -0.448 | -0.516 | -0.422 |
| Gly 143 | N | 0.181 | 0.321 | 0.340 | 0.199 | 0.207 |
| Gly 143 | CA | -0.461 | -0.386 | -0.139 | -0.193 | -0.181 |
| Gly 143 | C | 0.985 | 0.581 | 0.594 | 0.530 | 0.393 |
| Gly 143 | O | -0.324 | -0.499 | -0.477 | -0.400 | -0.468 |
| Ser 144 | N | 0.380 | 0.363 | 0.282 | 0.290 | 0.285 |
| Ser 144 | CA | -0.229 | 0.276 | 0.085 | -0.183 | 0.356 |
| Ser 144 | CB | -0.190 | -0.098 | -0.189 | 0.062 | -0.120 |
| Ser 144 | OG | -0.058 | -0.042 | -0.069 | -0.099 | -0.065 |
| Ser 144 | C | 0.335 | 0.278 | 0.221 | 0.586 | 0.318 |
| Ser 144 | O | -0.470 | -0.517 | -0.521 | -0.573 | -0.517 |
| Cys 145 | N | 0.403 | 0.553 | 0.270 | 0.236 | 0.447 |
| Cys 145 | CA | 0.689 | 0.397 | 1.013 | 0.063 | 0.857 |
| Cys 145 | CB | -0.509 | -0.542 | -0.932 | -0.665 | -1.122 |
| Cys 145 | SG | 0.321 | 0.421 | -0.552 | 0.305 | 0.847 |
| Cys 145 | C | -0.496 | -0.593 | -0.174 | -0.037 | -0.759 |
| Cys 145 | O | -0.411 | -0.451 | -0.432 | -0.490 | -0.463 |
| Gly 146 | N | 0.123 | 0.134 | 0.106 | 0.080 | 0.166 |
| Gly 146 | CA | 0.141 | 0.089 | 0.104 | 0.201 | 0.072 |
| Gly 146 | C | 0.500 | 0.550 | 0.531 | 0.440 | 0.572 |
| Gly 146 | O | -0.414 | -0.413 | -0.431 | -0.395 | -0.419 |
| EBS | C5 | -0.022 | -0.201 | -0.077 | -0.228 | -0.231 |
| EBS | C6 | 0.827 | 0.092 | 0.025 | -0.225 | -0.062 |
| EBS | C4 | 0.119 | 0.167 | 0.325 | 0.364 | -0.618 |
| EBS | C7 | 0.025 | -0.052 | 0.236 | 0.621 | 1.537 |

| | | | | | | |
|---|---|---|---|---|---|---|
| EBS | C(-CO) | -0.374 | 0.167 | -0.496 | -0.029 | 0.041 |
| EBS | C(-Se) | 0.727 | 0.179 | -0.702 | -0.791 | -0.141 |
| EBS | C3 | 0.521 | 0.130 | 0.210 | 0.812 | 0.084 |
| EBS | N2 | -0.129 | 0.540 | -0.415 | -0.740 | 0.341 |
| EBS | O | -0.389 | -0.388 | -0.071 | -0.458 | -0.307 |
| EBS | Se1 | 0.036 | -0.393 | -0.472 | -0.178 | -0.628 |
| EBS | C1’ | 0.005 | -0.999 | -0.399 | 0.112 | -0.578 |
| EBS | C6’ | -0.116 | 0.692 | 0.252 | 0.050 | 0.323 |
| EBS | C5’ | -0.142 | -0.302 | 0.028 | -0.077 | -0.348 |
| EBS | C4’ | 0.148 | 0.188 | -0.118 | 0.181 | 0.280 |
| EBS | C3’ | 0.137 | -0.110 | 0.793 | 0.295 | -0.038 |
| EBS | C2’ | -0.033 | 0.363 | -0.065 | -0.412 | 0.012 |
| EBS | O (2') | -0.094 | -0.050 | -0.497 | 0.039 | 0.000 |
| W1 | O | -0.058 | 0.048 | 0.371 | 0.039 | 0.037 |
| W2 | O | -0.099 | -0.366 | 0.622 | 0.037 | -0.147 |

**Table S10.** Energy values corrected including free energy (kcal/mol) in gas phase for the three used basis sets for the inhibition phase

| EBS | 6-31+G | 6-311+G(2d,p) | AUG-cc-pVTZ |
|---|---|---|---|
| EI | 0.0 | 0.0 | 0.0 |
| TS1 | 11.5 | 10.6 | 11.1 |
| EBS-OH | | | |
| EI | 0.0 | 0.0 | 0.0 |
| EI’ | -1.0 | -1.2 | -2.5 |
| TS1 | 11.1 | 8.2 | 8.7 |

## References


1 https://www.wwpdb.org/pdb?id=pdb_00006w63, 2020, .

2 Z. Jin, X. Du, Y. Xu, Y. Deng, M. Liu, Y. Zhao, B. Zhang, X. Li, L. Zhang, C. Peng, Y. Duan, J. Yu, L. Wang, K. Yang, F. Liu, R. Jiang, X. Yang, T. You, X. Liu, X. Yang, F. Bai, H. Liu, X. Liu, L. W. Guddat, W. Xu, G. Xiao, C. Qin, Z. Shi, H. Jiang, Z. Rao and H. Yang, *Nature*, 2020, **582**, 289–293.

3 L. Zhang, D. Lin, X. Sun, U. Curth, C. Drosten, L. Sauerhering, S. Becker, K. Rox and R. Hilgenfeld, 2020, **368**, 409–412.

4 R. Anandakrishnan, B. Aguilar and A. V. Onufriev, *Nucleic Acids Res*, 2012, **40**, W537-541.

5 Case, D. A.; Ben-Shalom, I. Y.; Brozell, S. R.; Cerutti, D. S.; Cheatham, T. E., III; Cruzeiro, V. W. D.; Darden, T. A.; Duke, R. E.; Ghoreishi, D.; Gilson, M. K.; Gohlke, H.; Goetz, A. W.; Greene, D.; Harris, R.; Homeyer, N.; Izadi, S.; Kovalenko, A.; Kurtzman, T.; Lee, T. S.; LeGrand, S.; Li, P.; Lin, C.; Liu, J.; Luchko, T.; Luo, R.; Mermelstein, D. J.; Merz, K. M.; Miao, Y.; Monard, G.; Nguyen, C.; Nguyen, H.; Omelyan, I.; Onufriev, A.; Pan, F.; Qi, R.; Roe, D. R.; Roitberg, A.; Sagui, C.; Schott-Verdugo, S.; Shen, J.; Simmerling, C. L.; Smith, J.; Salomon-Ferrer, R.; Swails, J.; Walker, R. C.; Wang, J.; Wei, H.; Wolf, R. M.; Wu, X.; Xiao, L.; York, D. M.; Kollman, P. A. AMBER 2017; University of California, San Francisco, 2017., .

6 J. A. Maier, C. Martinez, K. Kasavajhala, L. Wickstrom, K. E. Hauser and C. Simmerling, ff14SB, https://pubs.acs.org/doi/full/10.1021/acs.jctc.5b00255, (accessed 13 January 2021).

7 H. J. C. Berendsen, J. P. M. Postma, W. F. van Gunsteren, A. DiNola and J. R. Haak, *The Journal of Chemical Physics*, 1998, **81**, 3684.

8 T. Darden, D. York and L. Pedersen, *The Journal of Chemical Physics*, 1998, 98, 10089.

9 G. M. Morris, R. Huey, W. Lindstrom, M. F. Sanner, R. K. Belew, D. S. Goodsell and A. J. Olson, *Journal of Computational Chemistry*, **30**, 7.

10 J. Gasteiger and M. Marsili, *Tetrahedron*, 1980, **36**, 3219–3228.

11 G. M. Morris, D. S. Goodsell, R. S. Halliday, R. Huey, W. E. Hart, R. K. Belew and A. J. Olson, *JOURNAL OF COMPUTATIONAL CHEMISTRY*, **19**, 24.

12 C. I. Bayly, P. Cieplak, W. Cornell and P. A. Kollman, *J. Phys. Chem.*, 1993, **97**, 10269–10280.

13 N. A. Baker, D. Sept, S. Joseph, M. J. Holst and J. A. McCammon, *Proc Natl Acad Sci U S A*, 2001, **98**, 10037–10041.

14 E. Chovancova, A. Pavelka, P. Benes, O. Strnad, J. Brezovsky, B. Kozlikova, A. Gora, V. Sustr, M. Klvana, P. Medek, L. Biedermannova, J. Sochor and J. Damborsky, *PLoS Comput Biol*, 2012, **8**, e1002708.

15 J. Weiser, P. S. Shenkin and W. C. Still, *Journal of Computational Chemistry*, 1999, **20**, 217–230.
